# Endourological Options for Small (< 2 cm) Lower Pole Stones — Does the Lower Pole Angle Matter?

**DOI:** 10.1007/s11934-023-01161-w

**Published:** 2023-04-25

**Authors:** Angus Luk, Robert Geraghty, Bhaskar Somani

**Affiliations:** 1 Department of Urology, Freeman Hospital, Freeman Road, Newcastle-Upon-Tyne, UK; 2 Department of Urology, University Hospital Southampton, Tremona Road, Southampton, UK

**Keywords:** Lower pole angle, Narrative review, Ureteroscopy, URS, Percutaneous nephrolithotomy, PCNL

## Abstract

**Purpose of Review:**

Small renal stones in the lower pole are often difficult to treat. The angle of the lower pole to the renal pelvis (lower pole angle) is a limiting factor to rendering the patient stone free. This review explores the definitions of the lower pole angle, the various treatment options available, and how outcomes are influenced by the angle.

**Recent Findings:**

It is clear the lower pole angle definition varies widely depending on described technique and imaging modality. However, it is clear that outcomes are worse with a steeper angle, especially for shock wave lithotripsy and retrograde intrarenal surgery (RIRS). Percutaneous nephrolithotomy has similar reported outcomes to RIRS, and there is limited evidence it may be superior for steeper angles over RIRS.

**Summary:**

Lower pole stones can be technically challenging and adequate assessment prior to choosing operative approach is key.

## Introduction

Urolithiasis is a very common disease, with an estimated lifetime prevalence of around 10%, which is set to be increasing [1]. Kidney stone formation commonly takes place in the lower pole (35%), yet the endourological management of small lower pole stones remains a controversial topic [2••]. Treatment modalities for small lower pole stones include shockwave lithotripsy (SWL), retrograde intra-renal surgery (RIRS), and percutaneous nephrolithotomy (PNL). Each has its own benefits and drawbacks. There has been on-going effort to identify various factors which may impact the treatment outcomes of different modalities. The anatomy of the renal pelvis has been an area of particular focus, with the infundibulopelvic or lower pole angle thought to have significant influence on interventional outcome. This article will provide summarise the background of the lower pole angle, the issues surrounding its definition, how recent technological advances have impacted its relevance, and whether it still matters in the current day endourological practice.

## Defining the Lower Pole Angle

**Fig. 1:**
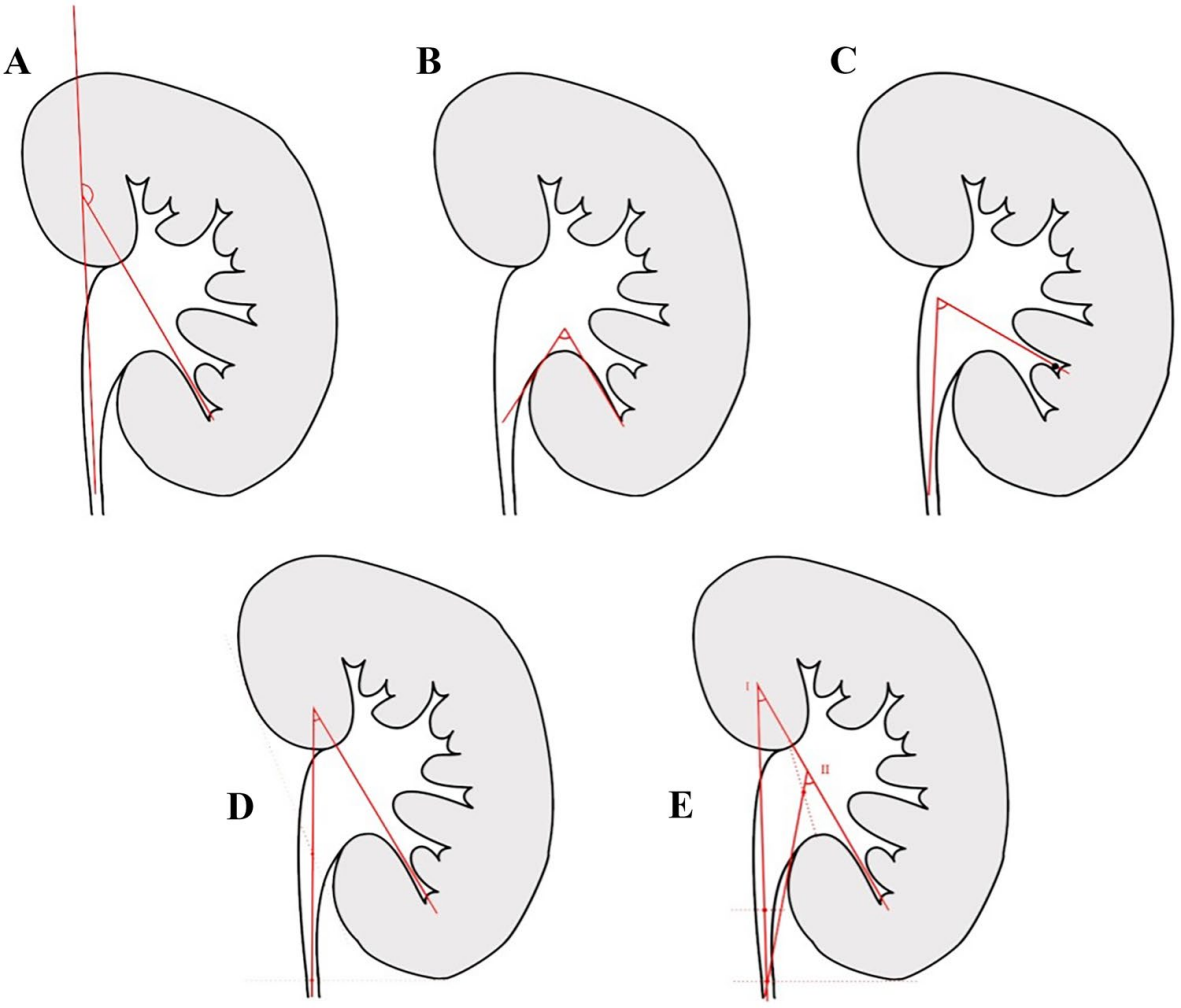
Diagram demonstrating different described methods of measuring IPA. **A** Bagley; **B** Sampaio (1992); **C** Sampaio (1997); **D** Elbahnasy; **E** Gupta

Several methods of lower pole angle measurements have been described in the literature (Fig. 1). The first mention of lower pole angle in the context of urolithiasis was probably by Bagley and Rittenburg in 1987, where they described what was termed the ureteroinfundibular angle (by measuring the major axis of the ureter to the axis of the lower infundibulum), to represent the deflection angle required in the context of designing flexible ureteroscopies (Fig. 1A) [3].

Sampaio and Arago first raised the possible role of lower pole angle in renal stone treatment efficacy in 1992; at the time, they simply described this as the angle between the lower pole infundibulum and the renal pelvis [4] (Fig. 1B). Sampaio et al. further refined the measurement method in 1997 by defining the infundibulum-pelvic angle (IPA) using two lines; the first line is formed between the central axis of the superior ureter (at level of lower pole) and the central axis of the ureteropelvic junction, with the second line being the central axis of either the major or minor infundibulum where the stone is located (Fig. 1C) [5].

In 1998, Elbahnasy et al. published an alternative measurement method of IPA, defined as inner angle form at the intersection of the ureteropelvic axis and the central axis of the lower pole infundibulum [6]. The ureteropelvic axis is delineated by a line was drawn connecting the central point of the pelvis opposite the margins of the superior and inferior renal sinus to the central point of the ureter at the level the lower kidney pole (Fig. 1D).

Gupta et al. [7] used 2 separate measurements of lower pole angle when looking at infundibulopelvic anatomy and SWL success rate [7]. They described using either the ureteral axis and ureteropelvic axis to form the angle measurement with the lower infundibular axis (Fig. 1E); both angles were associated with SWL success rate, and there was only a mean difference of 7° between them.

To date, there is no general consensus on which IPA measurement should be used [8]. No prospective comparative studies exists on examining which measurement method offers better predictive value on stone free rate. Manikandan et al. examined the IPA of a single lower pole stone-bearing kidney and the contra-lateral side, using the Sampaio, Bagley, and Elbahnasy method; only the Elbahnasy method showed statistical significance, implying this method could show predisposition to LP stone formation with respect to lower pole anatomy [8]. However, the difference between the mean IPA of the stone and the contralateral stone-free side was small (60.4° and 65.9°, respectively), and it is questionable whether this finding could be translated into determining which measurement method is best suited to guide management options. In the author’s experience, most reported literatures in recent years chose the Elbahnasy’s method in determining IPA.

The reproducibility of IPA measurements has also been called into question. Rachid Filho et al. examined the intra-observer and inter-observer variations of IPA measurements, with the Elbahnasy, Sampaio, and Gupta measurement method [9]. They found significant inter-observer variations, with the Sampaio method producing the widest variations between observers. This suggests that routine use of IPA in daily clinical practice may be problematic.

Historically, almost all studies looking at IPA in relation to lower pole stones utilised intravenous urogram (IVU) as its modality of imaging. The 2-dimensional representation of the renal pelvis anatomy could be significantly affected by several factors, such as rotation of the kidney in individual anatomical variations and positioning of the X-ray beam. Often the interpretation of IVUs may be difficult to due poor patient preparation [10]. Computed tomography (CT) has largely replaced IVU as the imaging of choice for urolithiasis in most current practices [1]; this also enables 3D-reconstruction of the urinary tract, giving a more accurate representation of the renal anatomy [9]. When using 30 degrees as a cut-off, one study found significant differences in IPA measurements between IVU and CT as imaging modality [11]. The lack of standardisation in imaging, where it is crucial to obtain accurate measurements, further hampers efforts to clearly define IPA and its role.

## SWL

It has been generally accepted that the efficacy of SWL is reduced for stones in the lower pole calyx when compared with other intra-renal locations, with the reported stone free rate (SFR) of SWL for lower pole stones varying widely between 25 and 95% [1]. Endourological procedures are considered to be a more effective treatment for small lower pole stones than SWL, particular for stones between 10–20 mm; nevertheless, SWL still has a role of being a non-invasive outpatient procedure; therefore, treatment options should be based on individual patient’s circumstances and preferences [12–14].

This is reflected in the EAU Urolithiasis guideline, where both SWL and endourological options are recommended for small lower pole stones [1]. The guideline does advocate for preference of endourological options over SWL in cases where there is unfavourable factors for SWL, one of which is a steep IPA. The exact threshold for when IPA would be considered “steep” is not specified; however; this is likely due to conflicting evidence in the literature, as well as the difficulty of defining where different methods of measurements exists.

The relationship between renal anatomy and success of SWL has long been under scrutiny. Sampaio originally proposed an IPA of < 90° as unfavourable for lower pole stone SWL, as he reported a SFR rate of 75% within the favourable group, compared with only 23% in those with IPA of < 90° [5]. Elbahnasy, using his alternate IPA measurement method, proposed an angle of < 70° instead; when combined with other unfavourable anatomy (infundibular width < 5 mm, infundibular length > 3 cm), the SFR was less than 50% [6].

Numerous studies have since showed a somewhat contradictory picture on the significance of IPA, with some suggesting it as a statistically significant factor, whilst others refute this, and often times suggested that other lower pole anatomical factors such as infundibular length and width were more important in determining SFR [15–19]. A more recent study by Chan and colleagues examining the efficacy of SWL in treating 10–20 mm lower pole stones suggested a statistically significant difference of the IPA between the success and failure group [20]. With the reported mean IPA of the 2 groups were 57.1° and 54.0°, respectively, it is difficult to see the clinical significance when there is such a small difference.

Few studies have directly compared different IPA measurement methods in SWL efficacy. Arpali and colleagues retrospectively analysed the radiological renal anatomy of patients who underwent SWL, with respect to their success rate; they applied both the Sampaio (1997) and Elbahnasy measurement for calculating the IPA in the same set of patients [21]. The mean IPA for all patients using the Sampaio and Elbahnasy measurement were 91.92° and 47.1°, respectively, suggesting that using different methods to calculate IPA can yield very different readings.

Overall, given the heterogeneity of measurement methodologies, high inter-observer variations, and conflicting evidence, attempts to determine a more precise relationship between IPA and SWL effectiveness remain elusive and may explain some of the conflicting study results. Indeed, it could well be argued that looking at IPA alone is perhaps not the correct approach; all other important factors such as infundibular length and width need to be taken into account, when determining whether the renal anatomy is favourable for SWL of the lower pole stone.

## RIRS

RIRS has largely overtaken SWL as the mainstay of nephrolithiasis management, largely owing to technological advancements in endoscope miniaturisation, improved optics, and deflection mechanisms [22, 23].

Despite this, treating small lower pole stones could be challenging with ureteroscopy (URS) due to hostile renal anatomy. A recent systematic review looking into the role of pelvicalyceal anatomy and SFR of lower pole stone treated with RIRS suggested that a steep IPA (Elbahnasy method) of less than 30° is the most significant predictor of being non-stone free following URS [24]. Infundibular width and length did not seem to significantly affect SFR.

The choice of flexible ureteroscope (fURS) should also be taken into consideration when tackling a lower pole stone in a steep IPA, as it would require the fURS to be able to achieve significant active and passive deflection in order to reach the stone. Whilst the diameter of the modern day digital and fibreoptic fURS are almost on-par, fibreoptic scopes tend to offer a slightly wider deflection angle which may give a slight advantage in demanding lower pole cases [25]. The tip of digital fURS is slightly larger and rigid in order to accommodate the digital camera chip, which often results in the loss of deflection at the distal tip for a few centimetres (termed end-tip deflection) [26]. An in vitro study using a RIRS training model tested 9 different fURSs their ability to access difficult angled calyx and the end-tip deflection in order to simulate difficult lower pole conditions showed that fibreoptic fURSs are generally better at accessing sharp angled calyx, as well as better end-tip deflection [27]. This study also highlighted that individual make of fURS all perform slightly differently, and it is important for endourologists to familiarise themselves with the equipments and choose the appropriate one for different settings.

A steep IPA is also associated with a higher risk of damage to ureteroscopes, which would incur significant cost to repair or replace [28]. With the recent widespread availability of single-use diposable digital fURS, they offer a breakage risk-free alternative when a high risk of scope damage is anticipated. Introduction of single-use fURS alongside reusable fURS has been shown to increase the life cycle of reusable scopes by 40% [29]. A recent meta-analysis suggested that their clinical outcomes are essentially comparable, and their cost is now considered to be similar to reusable fURS [30, 31]. The optical and technical characteristics of single-use fURS are generally thought to be somewhat inferior to reusable scopes, but this likely differs with individual makes, and it is debatable whether the difference is significant. Crucially in the context of lower pole stones, the deflection of single-use fURS was not found to be significantly different compared to reusable fURS [32].

The recent introduction of the thulium fibre laser (TFL) has permitted the use of smaller 150 µm laser fibres, instead of 200 µm fibres of the holmium:YAG laser. In the context of tackling small lower pole stones with a steep IPA, this offers several advantages. The smaller 150 µm causes less scope deflection loss, when compared with 200 µm and 272 µm fibres, where both had an additional 9 and 34 degrees of deflection loss, respectively [33•]. Scope deflection has been shown to reduce flow within the working channel, ranging from 2.9 to 9.4% depending on the make [32]. Having a device with smaller diameter within the working channel will reduce the impact on flow reduction, which may aid in reducing the possibility of thermal damage generated by laser use [34•]. Lastly, TFL has been shown to generate much finer stone dust when compared with holmium:YAG lasers, which may reduce residual stone fragments. A recent study using 3D printed kidney model showed that using TFL resulted in 35% less residual lower pole stone fragments when compared with holmium:YAG laser [33•].

## PNL

Historically, PNL for small lower pole stones had consistently produced the highest SFR out of all the treatment modality, at the expense of higher complication risks, greater pain and longer hospital stay. In recent times however, RIRS has produced almost comparable SFR due to maturation of technology, and there is still much debate in the endourology community on the best modality in tackling small lower pole renal stones [35]. The miniaturisation of PNL has off-set some of the drawbacks of this approach, whilst maintaining a very similar SFR [36].

There is conflicting evidence as to whether SFR and complication rates for small lower pole stones are higher with miniaturised PNL or RIRS [37•, 38]. On the other hand, it has been demonstrated that steep IPAs are associated with greater complication rates post-RIRS [28]. Factors such as prolonged operative time and increased intra-renal pressure intraoperatively may contribute to this. A recent study by Ozimek et al. reassuringly demonstrated that IPA was not associated with either SFR or post-operative complications following mini-PNL [39].

Huang et al. recently proposed a scoring system to aid in choosing between RIRS or mini-PNL (18-22Fr tract) for treatment of 1–2 cm lower pole stones [40]. The scoring system consists of 5 items, with one of them being the IPA (Table 1). A score of 0–2 or 3–5 would be advocated to proceed with RIRS or mini-PNL, respectively. This could prove to be a useful clinical decision-aid tool, but will require external validation.

**Table 1:** Scoring system proposed by Huang et al. to aid decision on endourological treatment modality for 10–20 mm lower pole renal stone

Factor		Points
Number of stones		
	Single Multiple	0 1
Stone diameter		
	≤ 15 mm ≥ 15 mm	0 1
IPA		
	> 90° < 90°	0 1
IL		
	≤ 30 mm ≥ 30 mm	0 1
IW		
	≥ 5 mm ≤ 5 mm	0 1

Total score of 0–2: recommend RIRS

Total score of 3–5: recommend PNL

*IPA* infundibulopelvic angle, *IL* infundibular length, *IW* infundibular width

## Conclusion

The role of IPA in treatment of lower pole stones has long been examined and debated. Yet to this day, there is still a lack of consensus on its definition/measurement method. Measurements of IPA are frequently found to not be readily reproducible accurately, and much of the original studies on the subject used outdated imaging modality. All these factors have made any meaningful, precise assessment on the role of IPA in lower pole stone treatment difficult and should be remedied in the first instance.

Despite this, there is little doubt that IPA still has a role in determining the most appropriate treatment option for small lower pole stones. It not only impacts the SFR, but also risk of complications, as well as choosing the most appropriate tool for the job. Whilst the current guidelines clearly favour RIRS/PNL over SWL in the presence of a steep IPA, the benefit between RIRS and PNL is not so clear-cut. With access to novel technologies only widely available in the last few years, such as TFL, suction, and PNL miniaturisation, it remains to be seen whether they will dramatically improve outcomes of their respective treatment modality in the presence of a steep IPA.
